# Ethnobotany in Intermedical Spaces: The Case of the Fulni-ô Indians (Northeastern Brazil)

**DOI:** 10.1155/2012/648469

**Published:** 2011-09-26

**Authors:** Gustavo Taboada Soldati, Ulysses Paulino de Albuquerque

**Affiliations:** ^1^Botany Post-Graduation Program, Biology Department, Federal Rural University of Pernambuco, Dom Manoel de Medeiros street, s/n, Dois Irmãos, 52171-900 Recife, PE, Brazil; ^2^Applied Ethnobotany Laboratory, Biology Department, Federal Rural University of Pernambuco, Dom Manoel de Medeiros street, s/n, Dois Irmãos, 52171-900 Recife, PE, Brazil

## Abstract

We analyzed the Fulni-ô medical system and introduced its intermedical character based on secondary data published in the literature. Then we focused on the medicinal plants known to the ethnic group, describing the most important species, their therapeutic uses and the body systems attributed to them. We based this analysis on the field experience of the authors in the project Studies for the Environmental and Cultural Sustainability of the Fulni-ô Medical System: Office of Medicinal Plant Care. This traditional botanical knowledge was used to corroborate the hybrid nature of local practices for access to health. We show that intermedicality is a result not only of the meeting of the Fulni-ô medical system with Biomedicine but also of its meeting with other traditional systems. Finally, we discuss how traditional botanical knowledge may be directly related to the ethnogenesis process led by the Fulni-ô Indians in northeastern Brazil.

## 1. Introduction

The present study consists of the authors' thoughts on their participation in the project Studies for the Environmental and Cultural Sustainability of the Fulni-ô Medical System: Office of Medicinal Plant Care. This project analyzed the medical practices of the Fulni-ô Indians (Águas Belas, Pernambuco, Brazil) and used local knowledge to improve access to health through the production of remedies based on plants. The present paper consists of an interdisciplinary proposal grounded between ethnobotany and anthropology—especially medical anthropology—and based on the interpretation of the authors as ethnobiologists. However, the positions defended here are entirely the authors' responsibility and do not represent the position of the team that participated in the project. The text is also based on a rereading of works previously published on this group [1–7].

First, we analyze local medical practices, above all the, therapeutic itinerary, which considers the different alternatives known and used by the Fulni-ô in their search for good health. Due to its multiple aspects, the Fulni-ô medical system will be analyzed using the concept of “intermedicality” that was originally used by Greene [8] to denote the hybrid and mutable nature of a shamanic system. In contrast to the widely accepted concept of static local medical practices, intermedicality results from the interaction between the local system and biomedicine. According to Greene [8], the specific practices show that the intimate relation between these two systems constructs a specific space that is contextualized and has values, symbols, practices, rites, and ethnological classifications to be incorporated, re-invented, and redesignated. 

Fòller [9] uses the same concept to study therapeutic practices among the Shipibo-Conibo. However, this author emphasizes a sense of conflict and struggle between the biomedical ideology (which represents the “colonial project”) and the local medical systems. Thus, these spaces of intermedicality not only create situations of interchange, as evidenced by Greene [8], but also political spaces of resistance against the hegemonic power and knowledge that biomedicine represents [9]. 

The concept of intermedicality has already been used to help understand the indigenous groups in northeastern Brazil. After investigating practices of healthcare access among the Ramkokamekrá of the state of Maranhão, Oliveira, [10] highlighted the power struggle between biomedicine and local medical systems. She also showed how the latter overlaps local practices. Silva [11] observed the same scenario among the Atikum of the state of Pernambuco and emphasized that biomedicine and traditional medical system “stand in opposition in the contest for legitimacy and for a privileged sphere of action (…) in a symbolic struggle for recognition within the community.”

We then investigate a specific part of this local medical system: the knowledge and use of medicinal plants. We describe therapeutic uses assigned to plant resources and bodily systems, and we describe the culturally most important plants based on the consensus on their use. We emphasize that this specific knowledge is also the result of different medical traditions and can be understood as “intermedical.” However, in spite of the fact that intermedicality has been defined as a “contact zone” that reproduces neocolonial discourse and Western ideology [8], we argue that the hybrid nature of the Fulni-ô pharmacopoeia contributes to greater local “medical security.” 

In addition to guaranteeing greater access to health, local knowledge on medicinal plants can be understood as an element affirming Fulni-ô identity. Fulni-ô pharmacopoeia is a component of an intermedical system and is used to discuss how this knowledge fits into and strengthens the process of “ethnogenesis” that is experienced by the indigenous ethnic groups in northeastern Brazil. We show that the process of ethnogenesis that is experienced by the Indians of northeastern Brazil also takes place at the local medical system. 

Our main goal is to use empirical and secondary data to analyze the Fulni-ô medical system as a space of “intermedicality” and “ethnogenesis.” Using these concepts, we assume that local medical systems are neither static nor mere reservoirs of pre-Colombian culture. Instead, they are creative and changeable, and thus this ability to *“actively negotiate the construction of their knowledge and practices is not a corrosive effect, but precisely what is necessary in order to assure their ‘cultural survival' ”* [9]. The present text supports the concept that Indians are active subjects in the process of sustaining their culture in spite of the historical difficulties they have experienced; we duly recognize Indians as “resistant” and not “resurgent” or “emerging.”

## 2. The Scene: Political, Historical, and Cultural Ethnicity of Fulni-ô Indians

The historical reality experienced by the indigenous ethnic groups of northeastern Brazil, which is strongly marked by an old and notable process of colonization, separates them from any attempt to apply existing generalizations about indigenous societies [12], such as the figure of the “Indian” that is widespread in the popular imagination. Similar to other indigenous societies of the same region, the Fulni-ô do not fit perfectly with the common imaginary view of the Indian [13]. The unity that exists between this ethnic groups is not shown through their social institutions or in their connection to the environment. Rather, the unity is shown because they have suffered and participated in the construction of northeastern Brazil as a political and historical “conglomerate” [14]. 

According to Oliveira [13], the specific reality experienced by the Indians of northeastern Brazil demands a different analytical basis originated from two specific aspects. The first aspect is associated with the existent pressure on the land as Northeast Indians seek to solve conflicts with landgrabbers, squatters, and large property holders regarding the use of the few arable lands in the region [14]. In contrast to indigenous groups in other parts of the country, such as the North, conflicts are tied to the demarcation of lands and the use of natural resources, such as ores and forest products. In addition to particularities linked to the land, the Indians of the Northeast do not have a strong cultural discontinuity and cultural visibility with respect to Brazilian society as a whole. The many years during which they were subjected to colonialist policies (persecution, discrimination, forced settlement, ethnocide, and subjugation) resulted in the absence of “diacritical” marks that would delineate different cultures. 

When one thinks of the northeastern indigenous people, in addition to recognizing their historical context, one emphasizes a phase of identity reconstruction or of the constitution of new ethnic groups who represent the protagonists for these societies based on a social process with a specific dynamic. Thus, indigenous communities of the Northeast are different because they have constructed a unique phenomenon of social reconstruction known as “ethnogenesis” (Ethnogenesis has also been called “journey back,” “emergence,” and “resurgence” [15].) This process, led by the Fulni-ô in the mid-1940s, is characterized by the political, historical, contextual, and situational (re)construction of a self-consciousness and a collective identity (and not only of a differentiation of culture and traditions) that is configured as a social limit with the purpose of achieving collective goals [14, 16, 17]. According to Oliveira [13], two factors favored this unusual process of “recuperation” by the ethnic groups: (1) economic and political contexts that strengthened the demand for the land and (2) the appearance of a favorable scenario for the old indigenous group recognition by the Service of Protection for the Indian (SPI). The old indigenous groups had their settlements officially abolished in the eighteenth century [13]. The principal demands were a differentiated state policy and access to land was fundamental to the necessities of subsistence and cultural reproduction. 

The first ethnogenesis events were unleashed by the networks of kinship existing among the indigenous groups that were not yet recognized by the state. Here, we strongly emphasize that the journeys of certain shamans and other Indians in these groups were a propulsive factor, which becomes a key aspect for the analyses proposed here. These journeys were crucial for the diffusion of politically constructed diacritical signs, such as the “tore.” This religious and sacred dance spread among groups and became a *“unifying and common institution (…) a political ritual, used whenever it was necessary to mark the frontiers between ‘Indians' and ‘whites'”* [14]. Distinctions were made evident by the religious aspects being modified, adapted, reinvented, or imported from other cultures during these events of mobility. Again, with respect to the importance of journeys and religions as ethnic affirmation, the same author states

*“... anthropologists know that pilgrimages can be important means for the construction of a sociocultural unity between persons with different interests and standards of behavior. (…) It is precisely this that is verified in the most recent studies on the ethnic groups of the Northeast. (…) We need to see that these journeys only took on this significance because the leader also acted in another dimension, making other journeys, which were pilgrimages in the religious sense, directed toward the reaffirmation of the moral values and fundamental beliefs that provide the bases for the possibility of a collective existence.”*

The Fulni-ô led the ethnogenesis events in northeastern Brazil, as they were the only group in the region that still expressed itself with its own language, Yatê (one finds different spellings in the literature when referring to the native tongue of the Fulni-ô, such as Ia-tê or Yaathê. We have chosen the form that appears in Rodrigues [18]), from the Macro-Gê trunk [18]. However, the Fulni-ô are further characterized by certain restrictions on marriage with non-Indians, by dancing the “toré” and by spending three months each year secluded in the village of “Ouricuri” to observe their sacred ritual by the same name. 

**Figure 1:**
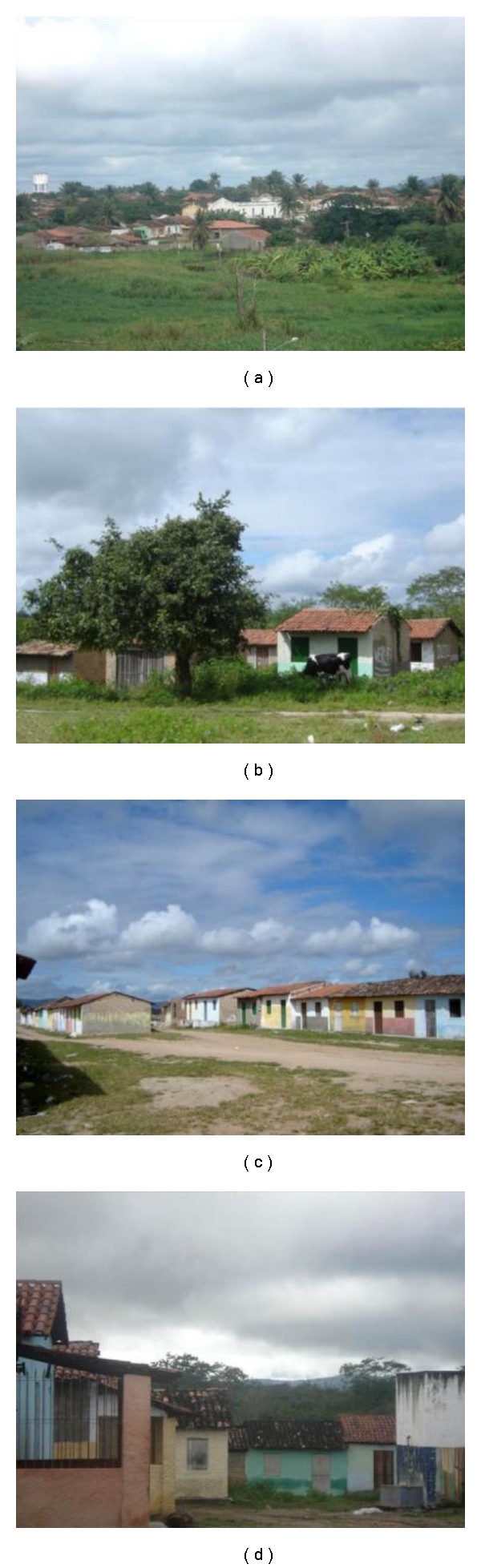
Fulni-ô Indigenous land, Águas Belas (NE Brazil). (a) “Main Village”; (b)–(d) “Ouricuri Village”.

According to oral tradition, the Fulni-ô, which means “those who live close to the river” (in this case the Ipanema River), is a fusion and settlement, on the part of the Portuguese crown, of five peoples who inhabited the region: Flowkassa, Tapuya, Brogadais, Carnijós, and Fulni-ô. At present, there are three villages in the Fulni-ô Indigenous Land: “Main Village,” where the great majority of the Indians live, and “Xixiaklá” and “Ouricuri,” where sacred rituals are observed (Figure 1). A total of 4336 Indians live in the Fulni-ô Indigenous Land (Terra Indígena Fulni-ô, TIF) [19], which is adjacent to the urban center of Águas Belas, separated only by a little creek. The main economic activities are agriculture, cattle raising, and handicrafts. Based on many visits to the village and many conversations with the Indians, including the shaman, we concluded that there is no basic sanitation and that trash is a recurring problem. 

**Figure 2:**
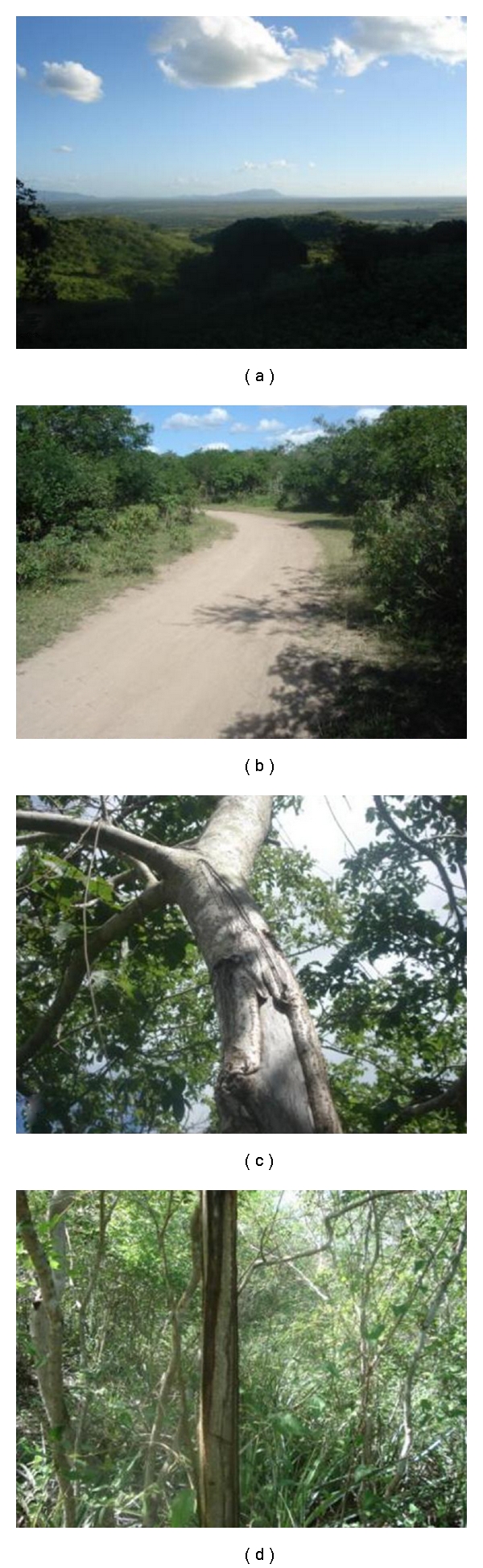
Fulni-ô Indigenous land, Águas Belas (NE Brazil). (a) Overall view of Fulni-ô Indigenous land; (b) road through the “Ouricuri Forest”; (c)-(d) bark extraction of medicinal plants in the “Ouricuri Forest” and its vegetation structure.

The Ouricuri ritual is an essential political and religious institution for the Fulni-ô and is important for a better understanding of the ethnic group and the analyses presented here. According to Souza [1], the Ouricuri *“is the principal ritual, and it plays an important role in social cohesion and in strengthening and giving value to the group identity and its religious perpetuation, thus becoming the thing most responsible for the symbolic configuration of the culture.” *The ritual is observed in the village of the same name, which is approximately seven kilometers from the main village. The Fulni-ô move there for three months of the year between September and November. The entire ritual, including its organization and prayers, is secret and is forbidden to non-Indians. However, some details are known. Communication is preferably in Yatê, which creates a space for passing on and perpetuating the language. During the Ouricuri ritual, the Fulni-ô intensely work on their spirituality not only because it is a time dedicated to prayers but also due to the proximity of the village to a native forest (“Ouricuri Forest”), which is considered to be the dwelling place of their ancestors (Figure 2). According to testimony, the months of Ouricuri are anxiously awaited because the ritual renews energies and creates spiritual peace. As good health is intimately associated with the Fulni-ô cosmology and religion, the ritual provides a special moment for healing (Laplantine and Rabeyron [20] hold that in these spaces for healing, there is a search for personal physical and spiritual well-being and for plenitude and wisdom. According to them, healing is situated “decidedly on the side of the sacred, and frequently of the secret (…), and we quickly cross the boundaries between that which in our culture is reserved for health, and that which is reserved for salvation”). 

Further, in the ritual, there is a spatial separation between the sexes. The women and babies live in little colorful houses usually located at the village periphery, while men sleep in a large central shed next to a “juazeiro” tree that is considered to be sacred. The consumption of alcoholic beverages, the use of electronic devices, and sex are prohibited. In the village of Ouricuri, there is no electricity or basic sanitation and trash is collected sporadically.

## 3. Data Collection

Some of the empirical data were collected by 344 semi-structured interviews between November 2007 and March 2008 from a stratified-probability sample of the Fulni-ô population inhabiting the head village, including only men and women over the age of fifteen (the procedures for which are detailed in Albuquerque [5, 6]). The project arose as an initiative of the ethnic group and the researchers became partners in the undertaking. The general interviews began with the presentation of the team and its objectives. The interviews lasted between 30 minutes and two hours, depending on those interviewed. Access to local channels and representations was facilitated by the presence of a native researcher, who was an important part of the team considering that questions could be associated with protected group secrets and its representations. This researcher's involvement allowed us to avoid needing a non-Indian researcher, who would likely be inexperienced in the culture and neglectful of social signs, taboos, and codes. This behavior could generate embarrassing situations and could risk not only the results of the research but also the researcher acceptance in the community (“in order to achieve real communication with those with other cultures, we need to discover etiquettes for intercultural communication. If “speaking” is important to communication, “not speaking” can have various meanings, from shyness and humility to disagreement or reprobation” [21]).

The team evaluation meetings were important for the identification of various problematic aspects, particularly regarding data recording. If a researcher is not completely acquainted with the nature of the questions for the instrument being used or is not trained regarding cultural questions, the researcher may record information in the wrong way and compromise the general interpretation of the results. This was the case for the immediate translation of some native categories. For example, informants cited plants for the treatment of “hemorrhoids.” In the local system, some people use the term to designate a type of parasitic infection. An immediate translation can generate information that is not trustworthy. Another case is that of the local category, “gastro,” which designates oral candidiasis in children (“sapinho”). The researcher, in an attempt to categorize, a priori, may translate the category as problems associated with the digestive apparatus. 

Our theoretical premises can justify the choices we made in terms of our methodological procedures. We started from the idea of culture as a system of shared knowledge [22]. Thus, a medical tradition based on the use of plants is part of a system of cultural beliefs and practices, which are shared and receive consensus among members of a group. 

To access local knowledge about medicinal resources, we opted for interviews with the use of questionnaires consisting of open-ended questions, which allowed room for a greater breadth of answers [23]. The application of questionnaires in a single-interview event was considered a limiting factor because some interviewees seemed not to recall plants used in their domestic practices at the moment of the intervention. However, when the sample of interviewees is representative, there is a good chance of recovering plants and information from the general consensus of the community, though at the expense of losing more specific information from the nuclear family. Thus, we did not assume that all interviews were individual because contributions from neighbors and family members were sometimes obtained, a process that could not be controlled by the interviewer. The presence of a fourth person at the interview may have motivated the interviewee to change his opinion or to furnish data that in principle did not belong to his domain. Depending on the context and the motivations, these interferences can affect the validity of the data collected [24]. 

 Questions specific to the ethnobotanical investigation focused on knowledge about plants: how they are used and prepared and the places where these resources are collected. Thus, the questions sought to account for the following information: (1) vernacular names of plants; (2) diseases (natural or supernatural) for which the plants were mentioned; (3) parts of plants used in preparations; (4) complete methods of preparation; (5) forms of administration of the medication; (6) quantities used in the preparation of the medications. 

For quantitative analysis, the citations from the interviews were examined and categorized at a later date. However, we tried as much as possible to respect the cultural specificities of the reality investigated. For example, in the case of “bálsamo,” we considered the following citations for a single ethnospecies: “bálsamo,” “baspo,” “basso,” “bássimo,” and “bássamo.” Similarly, therapeutic indications were later categorized, as was the case for “amidalite,” which brought together the following indications: “tonsillitis,” “tonsils,” “tonsils inflammation,” “inflamed tonsils,” and “tonsil pain.” To get an idea of the versatility of the Fulni-ô pharmacopoeia, its therapeutic indications (we refer to the native categories as “local therapeutic categories” or “local therapeutic indications,” which are culturally recognized diseases whether they are biological (in the sense of the biomedical tradition) or spiritual in nature) were categorized in 18 bodily systems according to the World Health Organization [25]: undefined problems or pains (AND); categories without biomedical correlation (CSB); diseases of the endocrine glands, nutrition and metabolism (DGE); infectious and parasitic diseases (DIP); mental and behavioral disorders (DMC); diseases of the skin and subcutaneous cellular tissue (DPS); diseases of the blood and hematopoietic organs (DSH); diseases of the osteomuscular system and connective tissue (DSO); pregnancy, birth and puerperium (GPP); injuries, poisonings and other occurrences from external causes (LEO); neoplasias (NEO); disorders of the sensory system (TOL); disorders of the sensory system (TOU); disorders of the circulatory system (TSC); disorders of the digestive system (TSD); disorders of the genitourinary system (TSG); disorders of the nervous system (TSN); and disorders of the respiratory system (TSR).

To get an initial idea about the most important plants in Fulni-ô culture, we developed an index for calculating “relative importance.” This index was constructed based on the study of various quantitative techniques used in ethnobotanical investigations [26] that aggregate different variables in a single instrument. In this sense, our index determines the “relative importance” of each plant based on (1) its versatility (wealth of uses reported); (2) total citations for the plant (an indirect measure of consensus about its use); (3) a correction factor (to avoid the overestimation of plants cited by few informants or the underestimation of well-known plants that are not very versatile). As shown above, this index reformulates existing techniques. We use it because we believe it is an adequate tool to analyze the reality investigated. However, as discussing the qualities of this new index is not the aim of this study, we will only do so in future paper.

The relative importance (RI) of each species was calculated by the following formula:

(1) $${\text{Relative}\;\text{Importance}}_{\text{sp}}=\text{Log}\left({\text{MU}}_{\text{sp}}+{\text{Cit}}_{\text{sp}}*\left(\frac{{\text{Ni}}_{\text{sp}}}{{\text{Ni}}_{\text{total}}}\right)\right),$$

where MUsp: wealth of medicinal uses of the species; Citsp = total citations for the species; Nisp = total informants who cited the species; Nitotal = total informants. 

In order to discuss the relationship between the Fulni-ô medical system and other traditional systems, all of the plants mentioned in the interviews were classified as native or exotic. Plants were considered native to the local medical system when their original geographic distribution could be traced to the South America. Conversely, plants were classified as exotic to the local medical system when their presence in the area was the result of human activity, whether intentional or unintentional. Thus, the plants considered exotic did not originally occur in the Brazilian semiarid, but were introduced by man. In this sense, it can be said that the exotic plants were introduced to the Fulni-ô through the contact with other cultures.

Finally, the team was also directed to collect the medicinal plants cited by the interviewees that were available in close proximity to the interview sites, such as in backyards, vacant lots, streets, or from neighbors, at the time of the interview. This procedure allowed for their scientific identification and avoided possible errors caused when extrapolations are made concerning the botanical identification (scientific name) of the ethnospecies cited. For example, *Ocimum gratissimum* L. is known locally as “alfavaca de caboclo” (caboclo basil), “alfavaca de vaqueiro” (cowboy basil), and “louro” (laurel). Another species, *Ocimum campechianum* Mill, is also known as “alfavaca de vaqueiro.” The collection of material while accompanied by each interviewee avoids the overestimation of the local importance of a botanical species that might happen if the record was based only on the popular name. If botanical materials were not collected for verification and material testimony, then every time “alfavaca de caboclo” was cited, for example, the association with one of these scientific names could incorrectly support the role of the species for the Fulni-ô. Likewise, the native research assistant was instructed on procedures for collecting botanical material with an emphasis on obtaining samples for scientific studies. This brought important benefits to the research: (a) the collection of specimens in a high quality of condition, which allowed for more precise identification and (b) the immediate manipulation of the samples (drying, pressing, and mounting) [27].

In the present study, out of respect for the Fulni-ô traditions and acknowledging the importance of local knowledge for the structure and differentiation of the local medical system, we will not present the confidential data or the list of species that compose the pharmacopoeia of the ethnic group. Moreover, the researchers signed a confidentiality agreement, and the publication of specific information about the plants has not yet been authorized by the ethnic group. The discussions presented here stem from theoretical reflection on information already available in the literature [1–7] and from the personal experience of the authors during their work with the Fulni-ô Indians.

## 4. Results and Discussion

### 4.1. The Fulni-ô Medical System: Knowledge and Use of Medicinal Plants

Within their medical system, the Fulni-ô use the medicinal properties of 243 native or exotic ethnospecies (Figure 4). Because we respect the confidentiality of the data, these are not presented here. Local remedies are prepared with preference for the perennial parts of the plants, such as bark and root. According to Albuquerque [28], this aspect of the use of medicinal plants, which is widely found in the Caatinga vegetation, may reflect an adaptation to the particularities of this semiarid region. However, the Fulni-ô also use other plant parts, such as fruits, flowers, and seeds, in making their remedies. The principal locations cited in the interviews for collecting resources were the backyards of interviewees' residences, the backyards of their relatives, the streets in Aldeia Sede, a mountain known locally as Serra do Comunaty (Mountain of the Community), and the native forest surrounding the village of Ouricuri. A total of 183 ethnospecies notably exotic species, such as “mentruz,” “hortelã da folha miúda,” “erva cidreira,” “samba caitá,” “capim santo,” “hortelã da folha grande,” and “boldo”—were collected in anthropogenic areas. The forest of Ouricuri, which is especially important during the ritual, offers native and arboreal resources, such as “aroeira,” “alecrim do mato,” “quixabeira,” “bom nome,” “imburana de cambão,” “imburana de cheiro,” and “juazeiro”. Thus, the resource zones play complementary functions in the local medical system by providing distinct resources. 

The Fulni-ô traditional medical system is quite broad and responds to a total of 18 bodily systems (Table 1). Including the *undefined problems or pains (AND),* the category that contains the greatest wealth of medicinal plants cited, there are 120 plants in total. This is followed by *disorders of the digestive system (TSD)* and *disorders of the respiratory system (TSR)*, with 98 and 91 plants, respectively. The therapeutic indications that had the greatest number of citations of use are colds, general inflammations, coughs, stomach pains, tranquilizers, fevers, wounds, strokes, wound healing, expectorants, and headaches. 

Using the index proposed in this study, the ten most important plants were: “aroeira,” “alecrim do mato,” “mentruz,” “sambacaitá,” “erva cidreira,” “quixabeira,” “hortelã da folha miúda,” “capim santo,” “bom nome,” and “imburana de cheiro”—of which five plants are native. In this list, “alecrim do mato,” “mentruz,” “sambacaitá,” “erva cidreira,” “hortelã da folha miúda,” and “capim santo” are exotic species. These ten plants also stand out with respect to the variety of bodily systems on which they work. That is, they are quite versatile. The “aroeira,” for example, is the most versatile plant with respect to bodily systems treated (14 in all), followed by “bom nome” and “quixabeira” (12 systems each). Other medicinal resources used are clays and fat from animals, such as tortoises, snakes and lizards, which are not plant-based but still deserve being reported.

### 4.2. The Fulni-ô Medical System: A Space of Articulation

The Fulni-ô medical system was characterized by Souza [3, 4] and mainly consists of two matrices that interact in the search for a cure: the traditional medical system and biomedicine—each represented by different institutions and agents. Specifically, according to Souza, the traditional Fulni-ô medical system is essentially shamanic, given that its conceptions about disease, health, and cures are intimately associated with religion and to the Ouricuri ritual. Knowledge and practices are transmitted orally and are concentrated in local specialists, called “rezadores” (prayers), “parteiras” (midwives), and “mais velhos” (elders).

**Figure 3:**
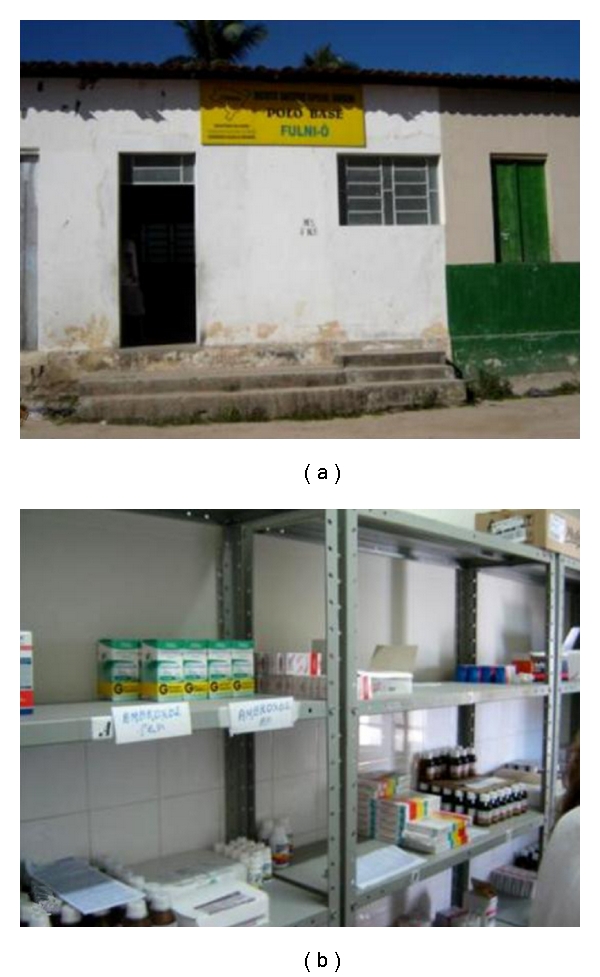
Base pole in Fulni-ô Indigenous land, Águas Belas (NE Brazil). (a) Base pole; (b) store of medications in the Base pole.

**Figure 4:**
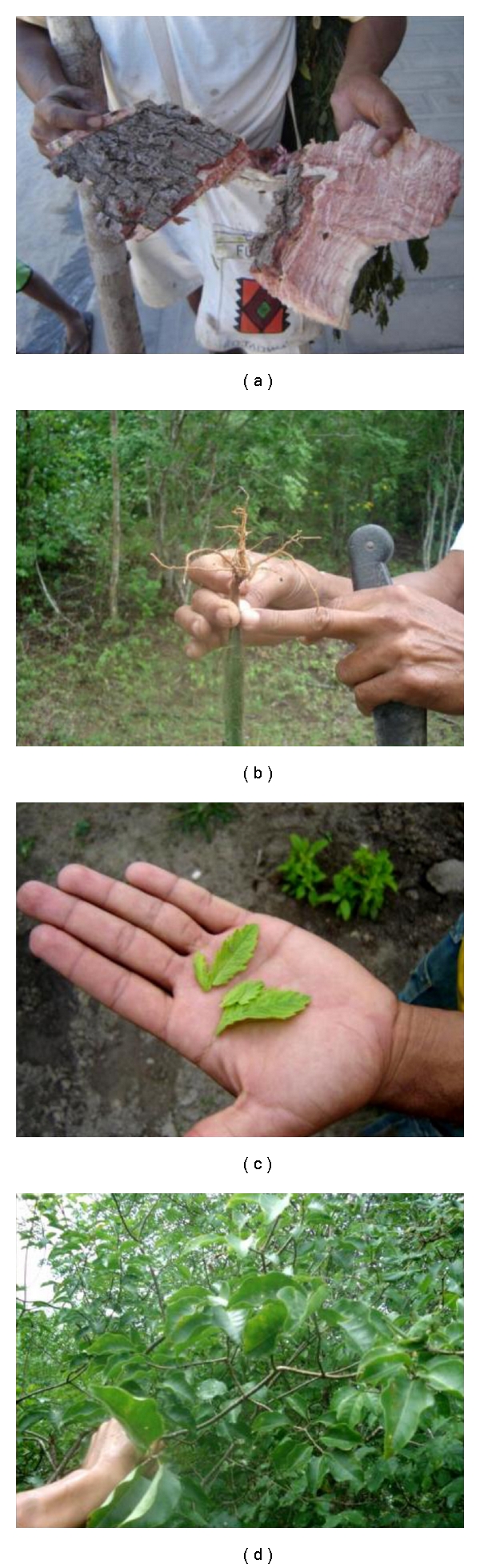
Use of medicinal plants in Fulni-ô Indigenous land, Águas Belas (NE Brazil). (a) A healer showing some medicinal plants collected in native forests; (b) a Fulni-ô indicating the part of the “urtiga” that is used in treatments; (c) “menstruz” leaves; (d) “pereiro”, a medicinal plant collected in native forests.

Biomedicine is represented in the Fulni-ô reality by the Base Pole (Figure 3), where one schedules appointments, exams and medical travel, and by the Health Center, where consultations, exams and dental treatment take place [2]. The Base pole is also responsible for the distribution of medications purchased by the DSEI/PE (special Indigenous Health District; for more details concerning the differential policy for indigenous health, see Athias and Machado [29]). To carry out specific activities, such as health campaigns, house calls, the scheduling of appointments, and the distribution of medicines for those who have problems with mobility, there is a team of Indigenous Health Agents (*Agentes Indígenas de Saúde, *AIS). According to Souza, when more special care is needed, such as surgery, the Fulni-ô receive biomedical attention in the Maternity of Águas Belas or are sent to large centers, such as hospitals in Garanhuns, Caruaru, and Recife. 

The Fulni-ô medical system shows clear evidence of its intermedical nature where a differential relation between power and capitalist ideology are present. The following are examples of intermedicality in the Fulni-ô medical system: the symbolic power and dependence of industrialized medications in the community; the high consumption of these same medications, with a turnover, in only one six-month period, of 120,000 Reais; the presence of a health center in the village of Ouricuri, the location of the sacred ritual, where there is the highest incidence of diseases and the greatest demand for traditional treatments, such as prayers and medicinal plants; the high demand for biomedical treatments even during the ritual of Ouricuri, in spite of the distance from the Base Pole; the appreciation of certain procedures and biomedical treatments, such as birth in the birth center, to the detriment of traditional practices, such as the role of “midwives”; symptoms and treatments for falling ill are linked to biomedicine, although explanatory models bring together different spheres of social and biological life, such as participation in the ritual of Ouricuri, the spiritual world, work, and emotional exhaustion; finally, poor service at large biomedical institutions, such as the birth center of Águas Belas. 

In addition, biomedicine is present in the practice of some of the Indigenous Health Agents who, although they are Indians, adopt the agency's discourse. In informal conversations, certain indigenous agents favored and gave value to biomedical practices in their activities. Fóller [9] found a similar situation and believes that this partial position is a “survival strategy” and not “opportunism,” as the local agents who were interviewed thought that the most appropriate technological package was the one that guaranteed their salary. However, in spite of the fact that Fulni-ô agents hold their positions by recommendation, the reason for their biomedical discourse still needs a better appraisal. 

Another example of intermedicality is the appropriation of biomedical categories to refer to symptoms and treatments. Various biomedical therapeutic indications and treatments were cited in the interviews, including those by local specialists. These included “colic,” “inflammation,” “antiseptic,” “depression,” “purgative,” “expectorant,” and “hypertension”. However, some biomedical terms were re-defined, as was the case for the “hemorrhoids” category that was initially understood by researchers as being “dilations of the veins of the rectum with or without flow of blood.” A better appraisal of the local meaning allowed us to observe that for the Fulni-ô, the local category “hemorrhoids” has to do with enterobiasis, a parasitic worm infection. Finally, we highlight names of certain plants used in traditional medicine that have associations with industrialized medications, such as “anador,” “dipirona,” “terramicina,” “ampicilina,” and “novalgina,” although this traditional/industrial overlap is not exclusive to the Fulni-ô experience. Albuquerque et al. [24] compiled information from the scientific literature on the use of medicinal plants by traditional communities in the Caatinga, and they demonstrated that this phenomenon exists in various locales. 

The examples cited above show clearly the intermedical field constructed in the Fulni-ô reality through contact between the traditional medical system and biomedicine, and some of these contacts show an asymmetrical power relationship. Capitalist ideology is explicitly marked by the creation and expansion of the local economy, showing, as Fòller [9] states, that the local acceptance of biomedicine cannot only be analyzed with respect to high technology and therapeutic efficacy. Local acceptance should consider economic and ideological factors as well. Contact between these two medical traditions influences the dynamics of the two systems but conceives points of conflict and clashes of interests that will make it difficult for traditional medicine to succeed. 

Thus, the examples cited above reinforce the concepts of Greene [8] and Fòller [9]: the intermedical field is a “contact zone” where points of conflict and clashes of interests are evident. Biomedicine represents a colonial project of domination that no longer takes place through economic or territorial conquest but happens through the strong influence on local cultures and, especially, through scientific knowledge. Despite the fact that this notion exists, the construction of an intermedical field may contribute to ethnic affirmation and to safer health access. In fact, the intermedical field begins with a relation of forces that are, in principle, asymmetric but that create a counter-force of resistance to power and hegemonic ideology. The counter-force unleashes resistance and cultural reaffirmation, which is a process of ethnogenesis. Thus, just as the demand for land released the first manifestations of ethnogenesis, the ideological pressures present in the Fulni-ô intermedical system are configured as a new sphere of demands for a differentiated ethnic identity. Finally, intermedicality diversifies the possibilities of finding a cure and optimizes access to health.

### 4.3. The Traditional Medical System as a Space for Ethnogenesis

To understand how the points of conflict and confrontation in the zone between the traditional medical system and biomedicine unleashed new forces for self-affirmation by the Fulni-ô—characterized by the desire to recover knowledge and traditional uses of medicinal plants—we will analyze the process that culminated in the planning of the project Studies for the Environmental and Cultural Sustainability of the Fulni-ô Medical System: Office of Medicinal Plant Care. We will also analyze the traditional botanical knowledge of the Fulni-ô Indians. This project is interdisciplinary and participative in nature and is financed and directed by the Area of Traditional Indigenous Medicine/VIGISUS II/FUNASA and carried out by the Mixed Association Cacique Procópio Sarapó (AMCPS). 

The recent journeys to the community, whether for cultural presentations or for the sale of handicrafts (especially in large urban centers), were fundamental for the process of construction of the Office Fulni-ô and for the first manifestations of ethnogenesis. Specifically, we highlight the role of José Francisco de Sá (Xycê, in the native language), the former president of the AMCPS, who traveled numerous times to Brasília (DF) looking for more profitable sales of Fulni-ô handicrafts. During one of his journeys, he was invited to participate in a course on medicinal plants, which strengthened his already existing interest in the therapeutic properties of plants. Considering the reality of the medical system, which is characterized by its precarious nature and excessive use of medications, among other conflicts, José Francisco learned about the existence of financial resources intended for research and promotion of traditional indigenous medicine. His initial project was approved by the National Health Foundation (Fundação Nacional da Saúde, FUNASA). His initial idea was to strengthen traditional practices—specifically the use of medicinal plants from the Fulni-ô pharmacopoeia—as an alternative to industrialized medications. According to Fòller [9], the awareness of traditions and identity is stimulated by direct contact with other realities. 

**Figure 5:**
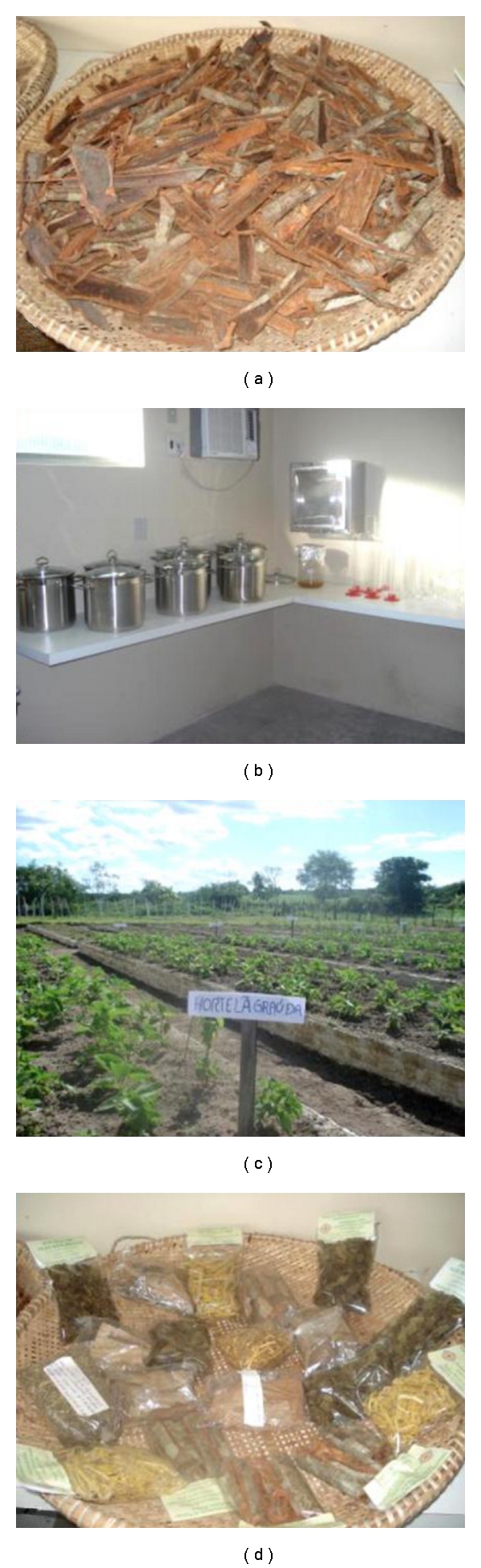
Fulni-ô office of Medicinal Plant Care in Fulni-ô Indigenous land, Águas Belas (NE Brazil). (a) Bark of “Aroeira,” a medicinal plant; (b) equipment for the production of herbal remedies; (c) bed and a nursery for the cultivation of medicinal plants; (d) herbal remedies.

The first advances were the construction of a bed and a nursery for the cultivation of medicinal plants at the TIF. Twelve Indians participated in a theoretical and practical course on management and associations, agricultural techniques, and the production of herbal remedies. A pharmacist offered training for the production of herbal remedies at a laboratory in Garanhuns, Pernambuco State. It was through these activities that José Francisco came into contact with plants that were still unknown to him, such as “poejo” and “transagem.” Later, when more funding became available, a laboratory for the production of herbal remedies and the Fulni-ô Office of Medicinal Plant Care was constructed with the appropriate equipment (Figure 5). Currently, the workshop is still preparing for production, but it is characterized as a space for sharing knowledge. Many Indians take some plants from its beds and have the opportunity to learn with José Francisco. 

One may conclude that concrete advances have been made in recovering and giving value to traditional knowledge. Even without the production of herbal remedies at the workshop, advances were made from the recognition of the importance of local practices of healing to the Fulni-ô identity and from recognition of the unfavorable situation of this medicine in confronting the domination of the discourse and practices of biomedicine. Like other activities, traditional medicine can be understood as resistance to an attempt to homogenize and subjugate an essentially heterogeneous and subjective system. 

The training of José Francisco in Brasília and the training of the 12 Indians in Garanhuns will allow for the production of herbal remedies as characterized by their scientific framework. Even the alternative of allowing a reduction in the consumption of industrialized medications is external to the culture. The forms of preparation, the locations and the tools are different from so-called traditional practice. The physical structure of the Fulni-ô Workshop for the Manipulation of Plants, with all of its equipment, is an institutionalization of medical practices (Figure 5). However, we need to make a distinction, as Fòller proposes [9], between biomedical “medicine” and biomedical “power.” In this sense, the presence of knowledge, practices, and technologies are recognized as ideological appropriations in which a process of “negotiation and renegotiation” in the construction and resistance of ethnic identity is “dynamic and transitory.” A similar phenomenon was identified by Greene [8], who identified the prescription of injections, or the complementary use of industrialized medications, in shamanic sessions among the Aguaruna Indians of Peru. The injections enchanted the Aguaruna and exhibited a high symbolic power for therapy, especially because of the character of the treatment—that of introducing into the sick person something that restores his or her health—is very close to their traditional explanations for cures and diseases.

### 4.4. Intermedicality, Ethnogenesis, and the Use of Medicinal Plants

The new understanding of the process of ethnogenesis of the Fulni-ô Indians—a cultural reaffirmation based on the value of traditional medical practices—also interferes with the utilization of some resources from the local system, such as medicinal plants. The Fulni-ô pharmacopoeia, the bank of medicinal plants known and used by the ethnic group, was considerably influenced by contact with other traditional medical systems, especially beginning with the historical experience of the Indians of northeastern Brazil, and once more, with the journeys made by members of the community. 

At least 102 (42%) of the 243 species that compose the Fulni-ô pharmacopoeia are exotic species. Assuming that an exotic plant was not originally part of the environment experienced by a given community, in our case, the region of the Fulni-ô Indigenous people, the incorporation of an exotic plant in the local pharmacopoeia occurs due to some contact with a different reality. In addition to detecting the presence of exotic species in the local pharmacopoeia, we also applied the index proposed. This index considers different variables jointly in order to detect the relative importance of plant resources in cultural practices of healthcare access. Thus, results showed that among the ten most cited plants, five are exotic. This information leads us to believe that in addition to being part of local knowledge, the exotic species play an important role in traditional practices due to their versatility and consensus about their use. 

This massive presence of exotic plants in the bank of plants used is viewed in different ways in the literature. Some authors state that their presence in the traditional pharmacopoeia is evidence of “cultural erosion” or “acculturation,” which reflects a passive vision of culture or a vision that is incompatible with the notion of ethnogenesis. In contrast, Albuquerque's [28] understanding of the importance of exotic plants for different cultures suggests that the presence of exotic species results from a process of “diversification.” The author understands that cultures are active in constructing their realities and that exotic species reflect a cultural “strategy” for diversifying the set of plants used, including new elements and making possible a greater range of useful resources. Albuquerque's [28] ideas agree with the Fulni-ô reality because the interviews showed that at least 14 therapeutic indications are treated only with exotic plants. 

As stated earlier, the lived history of the Fulni-ô influenced the construction of the current pharmacopoeia. Like all northeastern ethnic groups, they suffered various processes linked to the land, such as their sedentarization in villages, which aimed to homogenize them through catechisms and interethnic marriages [14, 30]. In some cases, like those of the Artikum of the Serra do Umã, different indigenous ethnic groups and groups of blacks inhabited the same portion of land [17]. The settlements or isolated territories became a space where different groups were able to share their traditional knowledge about cures—a process similar to what took place with the Fulni-ô. Many studies have investigated the presence of exotic plants in local pharmacopeias [31, 32] and have located intercultural exchanges as one of the principle motives [33]. For example, Janni and Bastien [34] studied the Kallawaya Indians in Bolivia and observed that a large part of their knowledge of medicinal plants comes from journeys by members of the various tribes to communities in different ecological zones. These authors state that a large part of the wealth of knowledge is due to the incorporation of knowledge developed externally to the Indian culture. 

Again, the role of travel in the enrichment of the local pharmacopoeia was fundamental, as in the case of Towê, the Fulni-ô Indian who has knowledge of many medicinal plants. Towê travels to different places, especially Brasília, to sell handicrafts and to publicize Fulni-ô culture through the Cafuia, a dance for artistic presentation. During these journeys, Towê has learned about different biomes and medicinal plants and has worked for a long time in a pharmacy with herbal remedies. As a result, of the 243 plants cited, 23 (9.4%) belong to his knowledge alone. Each trip brings something new to the Fulni-ô reality that can be incorporated into local practices if it passes through the local crucibles of the community. Journeys, thus, have the potential to enrich the local pharmacopoeia if the knowledge is shared with the community. Many studies have investigated the intracultural diffusion of knowledge [35–38] and note that many variables influence this process, including the social prestige of the individual who possesses the knowledge [39, 40]. That is, the knowledge learned and stored by Towé may potentially be absorbed by the entire ethnic group, but its diffusion depends on many variables, including Towé's political role and prestige within the Fulni-ô. 

The current Fulni-ô pharmacopoeia is the result of the incorporation of other knowledge, and thus the Fulni-ô medical system is plural—not only in offering different forms of access to health but also in being the fruit of many other medical systems. In spite of very notable traits of biomedicine found in local practices, the intermedicality of the Fulni-ô medical system is also constructed through the appropriation of other traditional systems from other cultural matrices (a phenomenon that shapes the pharmacopoeia for all the human groups in the Caatinga.) The appropriation and re-definition of these systems constructs the Fulni-ô identity at the present time and guarantees them, in spite of points of conflict and clashes of interests, various possibilities of access to cures. 

The intermedicality of the Fulni-ô system strengthens local “medical security” (an allusion to the concept of “nutritional security”). As documented by Souza [1, 4], many older people prefer to use traditional practices and seek services from biomedicine only when these are ineffective; in the same way, medicinal plants are an alternative cure for diseases that require expensive remedies, that are not available at the pharmacy of the Base Polo or that are difficult to access due to mobility problems. 

The existence of different alternatives for curing the same disease reduces the use pressure that is placed on the plants used in treatment, which contributes to the conservation of biodiversity. Albuquerque and Oliveira [41] evaluated this premise for a rural community in Pernambuco and observed that the use of a wide variety of species (i.e., alternatives) for the same therapeutic indications (headache, inflammation, fever) reduces the use pressure on each of the species and allows them a greater possibility of remaining in the environment for future use. In these cases, we hope that intermedicality will guarantee what we call “resilience” of medical systems. However, this scenario requires that locally used medicinal plants have the same prestige, which does not occur in the Fulni-ô reality. Interviews suggest that just a few species are highly preferred; that is, only a few species receive most of the attention in terms of collection, as is the case for “aroeira,” “imburana de cheiro,” and “alecrim do mato.” 

As noted earlier, the most cited therapeutic indications by the Fulni-ô were colds, general inflammations, coughs, stomach pains, tranquilizers, fevers, wounds, strokes, wound healing, expectorants, and headaches. These indications are exactly the ones treated with a greater range of species. In other words, the most frequent infirmities are treated with a wider spectrum of resources. Thus, we expect that this relationship will allow greater security in the treatment of the more frequent diseases, as many possibilities of treatment exist, which reduces the used pressure per species. However, future studies should be done to better evaluate this question.

## 5. Conclusions

Analysis of the Fulni-ô medical system, from the point of view of intermedicality, allows us to recognize its multiplex nature and the fruit of the hybridization of the local medical system with other traditional systems and biomedicine. Although there are well-defined spaces of action in each one of the traditions, given their proper specificity, there is an interaction with the construction of the local medical system that results in different points of articulation depending on the correlation of existing forces and the interests that are involved. We recognize that biomedicine is floating in an ideology that does not encourage heterogeneity but seeks homogenization as a means to domination. Nevertheless, its presence in the Fulni-ô reality strengthens their search for an identity and ethnicity and, with the traditional medical system as the driving force, allows for an outlet for another event of cultural reelaboration. Once more we see that the Indians are active agents in constructing their reality. The ideological appropriations presented here are not evidence of a cultural de-structuring but evidence of the incorporation of a symbolic power to renew forces, which guarantees cultural perpetuation. Oppression makes it necessary to struggle even against expressions like “resurgent,” “remaining,” or “mixed” Indians. As they themselves recognize, they are “resistant” Indians—which is the appropriate name for those who with much health, whether hybrid or not, have struggled against more than 500 years of cultural, economic, or political persecution.

## Figures and Tables

**Table 1:** Bodily systems addressed by the Fulni-ô medical system and the wealth of plants cited. Indigenous Land of the Fulni-ô, Águas Belas (PE).

Bodily system (WHO)	Wealth of ethnospecies
Undefined problems or pains (AND)	120
Categories without biomedical correlation (CSB)	23
Diseases of the endocrine glands, of nutrition and metabolism (DGE)	58
Infectious and parasitical diseases (DIP)	64
Mental and behavioral disorders (DMC)	37
Diseases of the skin and subcutaneous cellular tissue (DPS)	42
Diseases of the blood and hematopoietic organs (DSH)	11
Diseases of the osteomuscular system and connective tissue (DSO)	32
Pregnancy, birth, and puerperium (GPP)	23
Injuries, poisonings, and other occurrences from external causes (LEO)	60
Neoplasias (NEO)	13
Disorders of the sensory system (TOL)	5
Disorders of the sensory system (TOU)	14
Disorders of the circulatory system (TSC)	51
Disorders of the digestive system (TSD)	98
Disorders of the genitourinary system (TSG)	68
Disorders of nervous system (TSN)	22
Disorders of the respiratory system (TSR)	91
Not possible to report*	1
General Total**	843

*The information cannot be provided, especially due to cultural norms. **Refers to the set of all of the citations and not the total species cited, as it considers plants that were indicated for more than one system.
